# Youth and Provider Perspectives on Behavior-Tracking Mobile Apps: Qualitative Analysis

**DOI:** 10.2196/24482

**Published:** 2021-04-22

**Authors:** Courtney C Armstrong, Erica J Odukoya, Keerthi Sundaramurthy, Sabrina M Darrow

**Affiliations:** 1 Department of Psychology University of California, Berkeley Berkeley, CA United States; 2 University of Michigan Medical School Ann Arbor, MI United States; 3 Department of Psychiartry University of California, San Francisco San Francisco, CA United States

**Keywords:** qualitative, mHealth, mobile phone, behavior monitoring, youth

## Abstract

**Background:**

Mobile health apps stand as one possible means of improving evidence-based mental health interventions for youth. However, a better understanding of youth and provider perspectives is necessary to support widespread implementation.

**Objective:**

The objective of this research was to explore both youth and provider perspectives on using mobile apps to enhance evidence-based clinical care, with an emphasis on gathering perspectives on behavior-tracking apps.

**Methods:**

Inductive qualitative analysis was conducted on data obtained from semistructured interviews held with 10 youths who received psychotherapy and 12 mental health care providers who conducted therapy with youths aged 13-26 years. Interviews were independently coded by multiple coders and consensus meetings were held to establish reliability.

**Results:**

During the interviews, the youths and providers broadly agreed on the benefits of behavior tracking and believed that tracking via app could be more enjoyable and accessible. Providers and youths also shared similar concerns that negative emotions and user burden could limit app usage. Participants also suggested potential app features that, if implemented, would help meet the clinical needs of providers and support long-term use among youth. Such features included having a pleasant user interface, reminders for clients, and graphical output of data to clients and providers.

**Conclusions:**

Youths and providers explained that the integration of mobile health into psychotherapy has the potential to make treatment, particularly behavior tracking, easy and more accessible. However, both groups had concerns about the increased burden that could be placed on the clients and providers.

## Introduction

Problems with mental health are common among youth. Prevalence data suggest that 1 in 5 children has experienced symptoms of a mental disorder and 1 in 10 has experienced a serious emotional disturbance [1,2]. Mental health disorders that emerge during adolescence and early adulthood frequently continue into adulthood and contribute significantly to the existing global burden of disease [3,4]. Evidence-based interventions to treat mental illness have been developed and tested [5-7]. However, these interventions are not widely available, and of those who are able to receive treatment, younger clients are less likely to engage [6,8-11]. The integration of mobile apps into evidence-based interventions is one potential pathway to improving engagement and outcomes for adolescents and young adults (hereon, referred to as youth) [12]. As smartphone usage across all age groups has increased, the development and usage of mobile apps for the management of health has also increased immensely [13], and many mobile health (mHealth) apps are designed specifically for improving mental health [14,15]. In industrialized nations, the majority of youth have access to a smartphone or a tablet [16,17], thereby making apps increasingly accessible [18]. Prior research suggests that adolescents are interested in incorporating mobile apps into their treatment [19], and one study found that a mobile app that was integrated into treatment-as-usual with adults was effective and well received [20]. However, the initial promise of mobile apps has not been readily translated into widespread use. Despite their abundance, few apps designed specifically for youth are evidence-based [21]. Furthermore, previous research suggests that youth struggle to maintain long-term app usage [21,22], which can be a problem if attrition occurs before goals are reached or skills are fully learned. Given these considerations, there is a need for increased focus on user-centered approaches to mHealth [23].

Using mHealth apps with support from a health care provider has the potential to increase long-term engagement for youth [23,24]. Unfortunately, many health care providers are hesitant to integrate mHealth into their practice [25]. There are many factors that influence this, including poor fit to the treatment context or population and a lack of research exploring the perspectives of providers when it comes to integrating mHealth apps into routine care. Given that mental health interventions are unlikely to be adopted into practice or fully implemented if they do not fit the context of care, this is a significant barrier [26,27]. Thus, to achieve widespread implementation of mHealth apps, a better understanding of providers’ needs is imperative.

This study uses a qualitative approach to explore youth and mental health provider perspectives on using apps to enhance evidence-based clinical care, with an emphasis on gathering perspectives on behavior-tracking apps. Tracking behaviors, including mood and thoughts, is an effective component to many evidence-based treatments that leads to tangible changes in behavior and improved therapeutic outcomes [28]. Further, it is easily translated into an electronic task [29]. By exploring providers’ perspectives, this research highlights how mHealth apps could address the challenges providers face. Additionally, gathering the perspectives of youths who receive treatment will help identify the app features that will engage young clients. In-depth information on these topics may provide insight for future app designers, thereby ultimately improving the odds of successfully implementing an app to be used alongside face-to-face treatment.

## Methods

### Design

This was a qualitative, individual interview study to gather perspectives on mHealth usage from youth clients and providers. Interviews were conducted as part of an effort to develop a mobile app to be used alongside outpatient therapy for youth. Responses from the interviews were organized into codes and categorized into broader domains. All research activities were approved by the appropriate Institutional Review Board.

### Participants

#### Youth

A convenience sample of youths were recruited via study flyers posted within San Francisco. The inclusion criteria for participation were (1) between the ages of 13 and 26 years, (2) ability to speak English, and (3) participation in at least one session of outpatient psychotherapy. Youths were excluded if they had a visual, hearing, voice, or motor impairment preventing the use of mobile phones. Of the 10 individuals who contacted study personnel, all met the study criteria and were interviewed.

#### Providers

Recruitment emails were sent to providers working with youth at an academic medical center and to a listserv of community providers. The inclusion criteria for participation were (1) they currently conduct psychotherapy with youths between the ages of 13 and 26 years and (2) ability to speak English. Providers were excluded if they had a visual, hearing, voice, or motor impairment that interfered with use of mHealth apps. Of the 12 providers who contacted the study team, all met the inclusion criteria and were interviewed. The sample included 6 medical center providers and 6 community providers.

### Materials

#### Youth Interview Guide

Youth interviews consisted of 8 open-ended questions, followed by prompts to facilitate further discussion. Youths were asked questions regarding their preferences when selecting mobile apps, previous experiences tracking behavior during psychotherapy, app features that contribute to a positive user experience, and considerations that may facilitate the use of apps for tracking behavior during psychotherapy.

#### Provider Interview Guide

Provider interviews consisted of 5 open-ended questions, followed by prompts. Providers were asked to reflect on the outcomes of behavior tracking with clients and factors that affected the completion of behavior tracking. Prompts were used to determine the role of these factors in treatment-related decisions. Additionally, providers were asked to discuss current methods of behavior tracking, potential areas of improvement, and barriers to implementing electronic behavior tracking with clients.

### Procedure

Prior to the interview, participants completed an electronic consent form and a brief demographics survey using Qualtrics, a web-based survey platform. Telephone interviews were conducted to accommodate community providers; the remainder were conducted in-person at an academic medical center. Interviews took place in private spaces with no nonparticipants present. Repeat interviews were not conducted. Interviews were conducted by 1 of the 4 potential interviewers and were approximately 35 minutes in length (median 34.8 minutes, IQR 17.60 minutes). To begin, trained interviewers explained the purpose of the interview and reminded participants that they would be audio-recorded. Interviewers then audio-recorded and conducted the interview following the interview guides. Interviewers were asked to take notes during the interview process in order to assist with coding. Participants were emailed a US $30 electronic gift card. Interviews were conducted until no new knowledge was being obtained from the new participants.

### Data Analysis

Recordings were imported into the qualitative data analysis software Atlas.ti version 7 for coding and analysis. Two trained coders (EO and KS) reviewed each interview individually using a general inductive approach to analyze the data [30]. During the initial review, each coder listened for thematic content expressed by the participants related to the objectives of this research. Through discussion, the coders identified patterns in the data and created an initial code list to assign to portions of the recordings. During subsequent review, coders assigned codes originating from the previous discussions to quotations, and these quotations were transcribed. Coders met regularly to discuss discrepancies, develop new codes, and revise code definitions. As codes were further refined, interviews were continuously reviewed to adjust coding. Disagreements between the coders were resolved until complete agreement was reached by reviewing transcribed quotations and interview recordings.

## Results

### Participants in This Study

A total of 22 participants were interviewed for this study. This pool of participants consisted of 10 youths and 12 providers. The demographics of the youths and providers are illustrated separately in Table 1 and Table 2, respectively.

**Table 1:** Demographical data of the youths in this study (n=10).

Characteristic		Values
Age (years), mean (SD)		18.9 (3.73)
Gender (female), n (%)		9 (90)
Relationship status (single), n (%)		8 (80)
**Sexuality, n (%)**		
	Heterosexual	9 (90)
	Bisexual	1 (10)
	Homosexual	0 (0)
	Other	0 (0)
**Race/ethnicity, n (%)**		
	White/Caucasian	4 (40)
	Black/African American	1 (10)
	Asian	2 (20)
	Native Hawaiian/Pacific Islander	1 (10)
	Mixed race	2 (20)
**Residential environment, n (%)**		
	Urban	8 (80)
	Suburban	2 (20)
**Highest education level, n (%)**		
	8th grade or less	1 (10)
	Some high school	4 (40)
	Graduated high school or obtained general education diploma	1 (10)
	Graduated 4-year college	4 (40)
	Completed graduate or professional school	0 (0)
**Highest parental education level, n (%)**		
	8th grade or less	0 (0)
	Some high school	0 (0)
	Graduated high school or obtained general education diploma	1 (10)
	Graduated 4-year college	4 (40)
	Completed graduate or professional school	5 (50)
Currently in therapy, n (%)		8 (80)
**Length of time in therapy, n (%)**		
	<1 year	2 (20)
	1-2 years	1 (10)
	2-5 years	5 (50)
	5+ years	2 (20)
Taking medication for mental health, n (%)		8 (80)
**Employment status, n (%)**		
	Full-time employment	1 (10)
	Full-time student	5 (50)
	Part-time employment	2 (20)
	Employed, full-time student	1 (10)
	Unemployed	1 (10)

**Table 2:** Demographical data of the mental health care providers in this study (n=12).

Characteristic		Values
Age (years), mean (SD)		38.42 (5.78)
Years since received degree, mean (SD)		11.08 (3.8)
Proportion of time doing clinical work, mean (SD)		56.08 (30.65)
Proportion of clinical work with adolescents, mean (SD)		36.25 (20.35)
Proportion of clinical work with adults, mean (SD)		49.17 (25.03)
Gender (female), n (%)		8 (67)
Relationship status (single), n (%)		1 (8)
Licensed, n (%)		11 (92)
**Employment status, n (%)**		
	Full-time	10 (83)
	Part-time	2 (17)
**Race/ethnicity, n (%)**		
	White/Caucasian	9 (75)
	Black/African American	0 (0)
	Asian	2 (17)
	Native Hawaiian/Pacific Islander	0 (0)
	Mixed race	1 (8)
**Sexuality, n (%)**		
	Heterosexual	9 (75)
	Bisexual	1 (8)
	Homosexual	1 (8)
	Other	1 (8)
**Profession, n (%)**		
	Psychologist	6 (50)
	Social worker	1 (8)
	Licensed marriage and family therapist	5 (42)
**Theoretical background, n (%)**		
	Behavioral	1 (8)
	Cognitive behavioral	5 (42)
	Family systems	2 (17)
	Patient-centered	1 (8)
	Positive and self-compassion based	1 (8)
	Eclectic	2 (17)

### Youth Interviews

**Figure 1:**
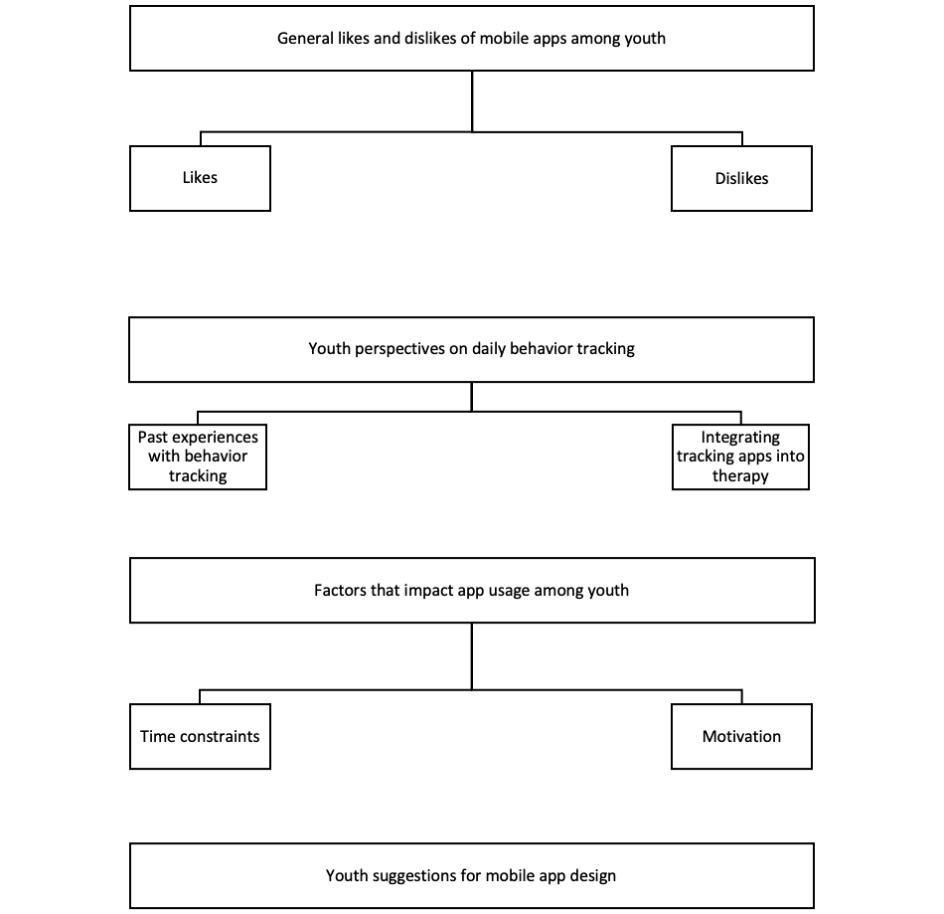
Summary of youth domains and subthemes.

Codes from the 10 youth interviews were sorted into 4 broad domains: (1) general likes and dislikes of mobile apps, (2) perspectives on daily behavior tracking, (3) factors that affect app usage, and (4) suggestions for mobile app design. These domains were broken up into subthemes. The domains and relevant subthemes are illustrated in Figure 1.

### General Likes and Dislikes of Mobile Apps Among Youth

#### Likes

All interviewed youths identified the mobile app features that they enjoyed. They identified 4 major positive features. The most commonly discussed feature was user-friendliness, which was defined by a participant as an app that “isn’t time-consuming or complicated to learn how to use.” [Youth 1] Other participants described enjoying apps that were easy to understand and made entering information a quick and efficient process. Youths also enjoyed receiving encouraging messages from apps and apps that helped them cope with stressors outside of therapy sessions.

#### Dislikes

All youths described negative experiences and the corresponding app features they disliked. They identified 4 major dislikes. First, the majority of the youths disliked apps that broke down frequently, (eg, issues with freezing, glitches, connectivity, other technical errors) preventing the app’s use. Second, a smaller group of participants reported frustration with apps that gave inaccurate or poorly organized information. These apps created more work and increased user burden. An example provided was auto-scrolling, which obstructs access desired to information. Additionally, participants disliked when information was obscured by advertisements. Third, another smaller group of youth disliked poorly formatted apps or apps that frequently changed their formatting, as this increased the effort expended to relearn how to use the app. Fourth, a small minority of participants expressed concerns with the lack of privacy; reasons included disliking any app that would not treat data with privacy and the ease with which others could find their information. Taken together, youth likes and dislikes strongly emphasize the importance of an attractive yet simple and consistent user interface that allows information to be made readily accessible.

### Youth Perspectives on Daily Behavior Tracking

#### Past Experiences With Behavior Tracking

Among the subset of participants who reported previously tracking behaviors as part of therapy, perspectives were mixed. Participants recognized that tracking behavior brought positive benefits to therapy, including improved quality of discussion with their providers. Additionally, tracking helped the youths set goals, monitor progress, and regulate behavior. However, the process of tracking behavior could be unenjoyable and anxiety-inducing for some participants. Additionally, 1 participant reported getting frustrated with the tracking over time despite positive first impressions. Another participant found tracking annoying but still perceived the benefits. Youths who had a positive or initially positive experience with tracking reported that they enjoyed the process and that tracking improved discussions with their providers. Among youths who disliked tracking behavior, a key commonality was feeling discouraged after entering information into a tracker and struggling to meet goals.

#### Integrating Tracking Apps Into Therapy

The youths gave mixed responses about using apps to track activities in therapy. Most provided reasons why using an app would be more beneficial than recording activities on paper. The reasons included increased convenience, more accurate tracking, improved privacy, more room for detail, and the ability to visually monitor progress with graphs and charts. A participant also noted that using an app to track activities would save paper. A smaller subset of the youth participants also shared reasons on why tracking activities with an app could be detrimental. They noted that tracking with an app was less personal and that the process of writing fostered a connection to what was being written down.

### Factors That Affect App Usage Among Youth

#### Time Constraints

Most youths reported that they would feel uncomfortable using their phones to track activities in certain situations (eg, at work, school, studying, with friends). One reported that this could be combated by setting time aside at the end of the day to record everything, which they preferred over monitoring activities throughout the day.

#### Motivation

A few youths noted that their own dislike for behavior tracking could lower personal motivation and make tracking harder, though behavior tracking would be easier if they considered it important. In comparison, a slightly larger subset noted that behavior tracking could be made easier by experiencing positive outcomes, such as providing content for therapy sessions and improved communication with their providers.

### Youth Suggestions for Mobile App Design

Youth participants suggested numerous features and considerations for a mobile app that would best suit their needs and facilitate behavior tracking. The most frequently made suggestion was that the app should have an interface that is both pleasing and easy to navigate. Additional features suggested by the youths can be found in Table 3.

**Table 3:** Summary of the suggestions provided by the youths for mobile health app features.

Function of feature, feature		Definition	Illustrative quotes
**Ease of use**			
	Reminders	Notifications reminding clients to complete their tracking	…*You’d want the app to send you some sort of notification. Like, ‘hey little check in: did this happen? Do you want to log it?* [Youth 9]
	Connecting to computer	Usable on multiple technological platforms	…*Something that can connect to my computer would be nice.* [Youth 7]
**Improve appeal of use**			
	Attractive design	Having an aesthetically pleasing and simple layout	…*You should be able to go in and not get frustrated while doing what you need to do.* [Youth 2]
	Customization	Ability to personalize activities and data input	…*I like how many options they have for tracking things. It makes it feel a lot more personal.* [Youth 5]
	Reinforcement	A feature that rewards activity completion	…*When you meet your goal, everybody wants a little pat on the back.* [Youth 3]
**Provide additional information**			
	Communication with provider	Enabled communication between client and provider outside of sessions	…*It would be great if there could be a messaging system where the therapist asks you questions about things that you say.* [Youth 4]
	Output to client	Organize/summarize data for easy review	…*Charts are important because it’s a visual way to see the progress you've made or if you're slipping.* [Youth 1]
	Additional resources	Clients or providers able to add relevant information to be accessed between sessions	…*It would be great if skills you used could be saved.* [Youth 4]

### Provider Interviews

**Figure 2:**
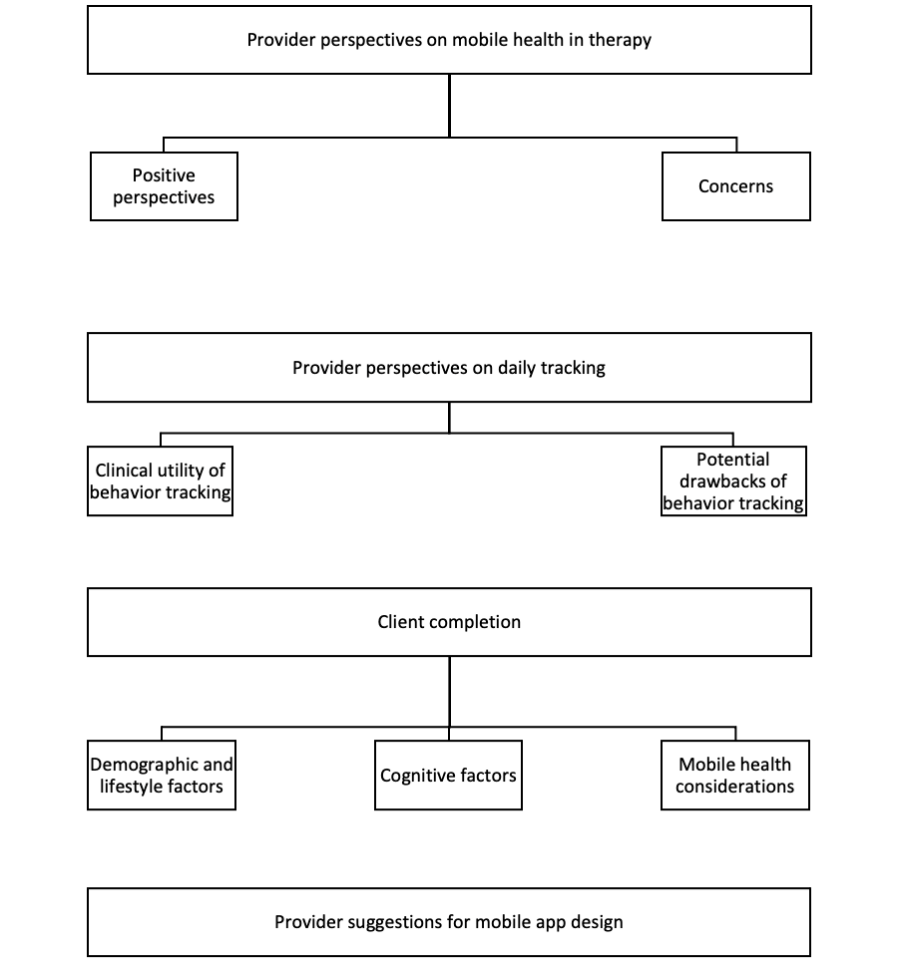
Summary of provider domains and subthemes.

The codes from the 12 provider interviews were categorized into 4 umbrella domains: (1) perspectives on mHealth, (2) perspectives on behavior tracking (3) client compliance, and (4) suggestions for mobile app design. These domains were broken into subthemes. The following sections present qualitative data from the provider interviews describing these subthemes (see Figure 2 for overview).

### Provider Perspectives on mHealth in Therapy

#### Positive Perspectives

The most frequently mentioned benefits to the client included that apps are easy to use and more attractive to younger clients when compared to pencil and paper. Two providers reported that it would be useful for clients to frequently provide updates on their lives in the moment. Nearly all providers discussed ways in which electronic behavior tracking could make their clinical work easier. Primarily, these providers spoke of how behavior-tracking apps could summarize trends in client behavior by using an organized format.

#### Concerns

Providers reported 6 primary concerns related to the use of electronics in clinical practice. Most frequently, providers reported that behavior-tracking apps can be confusing and that “if [an app is] not very self-explanatory or not very easy to access, [clients] won’t do it probably.” [Provider 3] Half of the providers also expressed that they were worried about apps being inaccessible to clients either due to Wi-Fi and connectivity issues or for financial reasons. One provider voiced discomfort with the idea of increasing their clients’ use of technology and suggested that writing instead of typing would help clients better process information. Some providers were concerned that incorporating technology into their practice would lead to unnecessary additional work and frustration. They noted that receiving too much information too frequently can be overwhelming and mHealth apps might have problems with security or assume a level of technological understanding that not all providers have. Additionally, the notion that electronic tracking needs to have clinical utility was highlighted, “If the data are just going to a data bank, it’s kind of static, just like paper...then maybe it’s not necessary for tracking to be tech-based.” [Provider 12]

### Provider Perspectives on Daily Tracking

#### Clinical Utility of Behavior Tracking

Providers noted that behavior tracking provided important information about clients’ behaviors and can facilitate behavior change by increasing awareness of relevant behaviors, thus benefiting both them and their clients (eg, “It's useful for identifying triggers or identifying thinking patterns” [Provider 4]). Some providers shared that behavior tracking was also useful for improving communication, specifically stating that behavior tracking allowed them to make therapy more personal and helped clients set session agendas.

#### Potential Drawbacks of Behavior Tracking

Providers reported several drawbacks related to behavior tracking. Although individual responses varied, all were related in some way to client completion. A few providers stated that clients often failed to track activities every day, leading to less accurate information. One provider stated that when behavior tracking was incomplete, valuable session time was spent on problem solving. Additionally, some providers stated that tracking every day can feel burdensome to clients, with one also stating that activities done outside of the session can be tracked incorrectly. This is particularly relevant in the context of family therapy, as providers described how difficult it could be to manage daily behavior tracking for multiple people.

### Client Completion

#### Demographic and Lifestyle Factors

Half of the providers reported that older clients are generally more willing to track behaviors outside of session. This was encapsulated by the statement, “Age does help, and the ability to understand the potential utility of [behavior tracking] as a strategy.” [Provider 1] Half of the interviewed providers also reported that clients with a higher socioeconomic status were more likely to complete daily behavior tracking, as “lower socioeconomic status clients are more in survival mode, and that will often trump everything else.” [Provider 3] Providers also reported specific factors such as lifestyle, education, and family structure were likely to impact completion. Clients who are able to give more of their time and energy to tracking an activity in daily life are more likely to complete. To quote one provider: “Clients where I have seen it not work...have multiple other life burdens in terms of their hierarchy of needs.” [Provider 5] Similarly, having family structure and consistency in the household was thought to increase the likelihood of a client tracking daily behaviors. Clients who are in school, complete daily homework, or complete other daily routines were also suggested as more likely to track behaviors consistently. One provider reported race as a potential factor affecting client completion, although specific racial groups were not mentioned.

#### Cognitive, Behavioral, and Personality-Related Factors

Cognitive, behavioral, and personality-related traits that providers suggested would decrease the likelihood of clients completing daily tracking included issues with attention, motivation, memory, pathology, shame, and nonspecific cognitive difficulties. Specifically, clients who experienced trouble with attention or memory were thought to be likely to struggle with tracking due to difficulties with completing the behavior of interest. Clients who experience difficulties with motor function or vision could have trouble completing a tracking log, and clients who could not comprehend the activity being tracked would be unlikely to complete it. Clients who experience significant shame, specifically related to the behavior being tracked, might also be unwilling to report instances of the behavior to a provider.

Cognitive, behavioral, and personality-related traits positively affecting completion included clients described as responsible, anxious, or having positive behaviors, and feelings related to therapy generally. A few providers reported that “responsible” clients, and clients who were more “rules-oriented” were more likely to complete therapy-related tasks, particularly behavior tracking. Additionally, those who have more trust in their provider or exhibit more buy-in regarding therapy are more likely to complete assignments. Three clinicians explicitly mentioned that youths who had sought out therapy themselves, rather than having parents seek out treatment on their behalf, are more likely to complete tracking.

#### mHealth Considerations

Providers reported 8 ways that mHealth apps could be used to improve completion. Specifically, providers said that an app would make it easier for clients to complete the activity. Half of the providers thought an app could be used to set up systems of accountability, ensuring that clients complete the assigned activity. Some reported that apps could remind clients to complete tracking activities, although there was some concern that clients would habituate to reminders over time. Providers also reported that apps could provide examples to improve understanding of tasks, pair assigned activities to more enjoyable activities to increase motivation, make assigned activities seem more relevant to the client, make the tracked activities seem less rigid, and reinforce completion.

#### Provider Suggestions for Mobile App Design

Providers suggested 25 different types of behaviors that they might want to track with an app (Textbox 1).

Providers also suggested multiple features for a behavior-tracking app that would best suit their needs. The most frequently given suggestion was that an app should clearly summarize client data. Additional features suggested by the providers can be found in Table 4.

**Behaviors to track that were suggested by the providers.**

- Physical activity
- Emotional intensity
- Conflict
- Device use
- Substance use
- Sleep and appetite
- Attendance
- Irritability
- Problem behaviors
- Social activities
- Thoughts
- Urges
- Community and citizenship
- Fatigue
- Motivation/concentration
- Relationships
- Relaxation activities
- Therapy homework
- Work/school
- Achievement
- Creativity
- Driving
- Money use
- Sexual risk taking
- Extracurricular activities

**Table 4:** Summary of the provider suggestions for mobile health app features.

Function of feature, feature		Definition	Illustrative quotes
**Ease of use**			
	Simplicity	Having an intuitive and efficient interface	…*Something super intuitive that could be used in a way that is almost dummy-proof. So even if you’re not super tech savvy… it would just be so easy. And quick.* [Provider 6]
	Reminders	Notifications reminding clients to complete their tracking	…*A reminder is always helpful… I think for some people, an alarm or a popup, a sound, or whatever would be easy for people to see and do. They can just click it and go right into it…being able to set up when and how it shows up, according to the* *patients'* *preferences.* [Provider 7]
	Remembering data	Previous entries remembered to ease data entry	…*It would remember what I'd already entered and add it to the menu, so if I had the same breakfast three days, I could just hit breakfast and it would just put in all three.* [Provider 10]
	Data-based suggestions and advice	Tips or information about the tracked behavior	…*Kind-of a help button. Any kind of monitoring form can be confusing… when we ask about your mood scales, there's kind of a definition with each question… it's really clear what information is needed.* [Provider 12]
**Improve clinical utility**			
	Customization	Ability to personalize activities and data input	…*My dream application would be a sophisticated application that allows you to create scales and create the flexibility to make whatever scale I want. So, it’d have a bunch of different ways to track something*. [Provider 11]
	Provider notifications	Alerts telling the provider when the client enters data	…*[Something that] gets communicated to me via link or email and it’s just very clear and understandable and it doesn't take too much time for me to understand it.* [Provider 1]
	Output for providers	Organized summaries of client data such as a table or graph	…*If there were a system… that automatically graphed things for you… So I could log in as their provider and see a graph of progress over time.* [Provider 1]
	Parent information	Allow parents to enter information	…*Maybe it would be nice if there was a child feature and a parent* * feature, or if there was an ability to link between phones so the parent and the child could input data.* [Provider 8]
**Improve appeal of tracking**			
	Visually appealing	Pleasing interface rather than plain text	…*Something that reminds the client to take some time and sit down… I wouldn't want the app to be like opening up the notes section of your iPhone.* [Provider 5]
	Reinforcement	A feature that rewards activity completion	…*[There should be] immediate reinforcement on completing it.* * Technology has built up youth's dependence on instant gratification, so using that in a way that would help them continue to be using it.* [Provider 8]
	Options	Multiple-choice options for describing mood, rather than an open-ended format	…*Being able to scroll down and select [from a list of] cognitive* * distortions.* [Provider 2]
**Provide additional information**			
	Output to clients	Data summaries that are accessible to the client	…*If the app can output the data back to the client in a way that helps them see like, ‘oh on average you’re getting so much sleep!’… Because that's what monitoring is about. To help us better understand these patterns, and make use of these patterns, to make use of it in therapy and treatment and address it.* [Provider 7]
	Crisis resources	Provide crisis resources	…*What would be great is having a page or a tab, or something where there's a list of local crisis resources, so text line, lifeline, rape line, so somehow pulling from crisis stuff.* [Provider 9]
	Social support	Provide social support or connect to social media	…*It lets me post to social media, so you get that kind of support too.* [Provider 7]

## Discussion

### Principal Results

This study explored client and provider perspectives on using mHealth apps to enhance clinical care and identified features that they would want in an app designed for daily behavior tracking during clinical care. This study adds to a growing literature exploring provider perspectives on mHealth and is the first to do so while synthesizing youth and provider perspectives, thus improving our understanding of mHealth’s potential to improve behavior tracking’s accessibility and usage. Both providers and youths considered behavior tracking to be a beneficial activity that led to more meaningful therapy sessions and behavior change. These findings are consistent with the broader literature showing that tracking behavior helps clients maintain healthy attitudes, counteract negative developments, and improve self-management [31]. Furthermore, supplying providers with additional information regarding a client’s behavior can improve treatment outcomes [32]. Reasons for this include the possibility that additional information from clients allows providers to tailor session content based on events that occur outside of therapy [31]. Youths and providers both believed that using an app could be a more enjoyable and accessible way to track behavior. Widespread smartphone usage facilitates behavior tracking in situations where clients cannot access paper-based resources used to supplement many mental health interventions (ie, diary cards, handouts, and workbooks). This could increase the accuracy of behavior tracking, further improving clinical utility. Additionally, apps have the potential to summarize collected data in a format that is easy to understand, making progress-tracking easier and facilitating discussion of progress during treatment.

Despite the recognized benefit of daily tracking, views regarding completion and app usage were mixed. Select youth participants found behavior tracking frustrating, which could impede behavior tracking. Providers also believed that negative emotions could hinder behavior tracking, particularly for those too young to fully appreciate its long-term benefits. Youths and providers were also concerned about the added burden of an app. Youth participants disclosed that social environments (eg, school, work) limit opportunities to track behaviors. Likewise, providers stated that individual pathology or factors related to socioeconomic status might negatively impact clients’ completion of behavior tracking. To minimize burden on youth clients, providers and youths suggested that an app should be highly customizable so that only relevant information is tracked. It should be noted that many of these challenges related to burden parallel what clinicians already encounter with paper-based tracking.

This problem of increased burden is not solely applicable to clients and highlights a notable way in which client and provider preferences may conflict. Indeed, half of the youth participants wanted an app that would allow for communication with their providers between sessions. However, providers expressed concern that an app could be overwhelming due to increased information received from clients and insufficient understanding of technology. This is a significant barrier to the integration of mHealth into routine care, as evidence-based interventions are difficult to implement when providers lack sufficient training, resources, and support [33]. To circumvent this issue, app designers should prioritize minimizing burden placed on both providers and clients. Features such as reminders and more immediate reinforcement may be helpful for some clients, but they may not work for others. Additionally, some providers may want to communicate with clients via an app, while others may not. Designing a flexible behavior-tracking app that allows for customizations as well as considering how a platform could be used in the case that paper-based tracking will be preferred by some, may be the best solution for widespread implementation. Youths and providers also had concerns about data security and privacy. The use of passwords and secure login portals are potential solutions suggested by both providers and youth. Prior research highlights additional steps that can be taken to ensure client data are secure [34].

### Strengths and Limitations

This study is among the first to compare youth and provider perspectives regarding the integration of mHealth into evidence-based, routine psychotherapy practice. Taking the perspectives of both groups allows future researchers and developers to consider the best possible ways of meeting the needs of both groups without placing inhibitory burdens on either. Regular review of the recorded interviews and interview guides allowed for a thorough examination of participant-driven topics. There are limitations to this research. Because the mean age of the youth participants was 18.9 (SD 3.73) years, their perspectives might not fully reflect the views of younger adolescents. Additionally, the youth participants in this study were 90% (9/10) female. However, this is consistent with that reported previously in treatment-seeking populations [35]. Despite our relatively small sample size, participants shared a variety of challenges to implementing an mHealth app as well as many different behaviors that might be clinically indicated for different individuals to track.

### Future Directions

This study highlights provider and client perspectives on the acceptability of using behavior-tracking apps in clinical practice. Additionally, the study discusses design features that would better facilitate widespread implementation of behavior-tracking apps. Specifically, providers and clients alike stressed the need for features such as reinforcement, which may assist with long-term use. Flexibility and simple interface design were also deemed important and could help to minimize the burden placed on providers. Future research should explore whether specific features such as reinforcement and interface design reduce the perceptions of burden and facilitate user engagement. Additionally, it may be beneficial to examine the feasibility of tracking apps across specific modalities and treatment environments such as community mental health clinics and private practice.

### Conclusions

This study shows that mHealth has the potential to improve daily behavior tracking for youth recipients and providers of mental health services. First, this study highlights that behavior tracking is generally acceptable to both youths and providers, largely due to the increased information shared between clients and providers. Second, it explores ways in which mHealth can make behavior tracking more accessible and enjoyable for both clients and providers. Nevertheless, to achieve widespread implementation, future development and implementation efforts must pay special attention to a potential app’s ability to meet the individual needs of providers and youths without placing overwhelming burdens on either.

## Abbreviations

mHealth: mobile health
